# Severe Unexplained Relative Hypotension and Bradycardia in the Emergency Department

**DOI:** 10.1155/2014/969562

**Published:** 2014-03-05

**Authors:** Shivam Kharod, Candice Norman, Matthew Ryan, Robyn M. Hoelle

**Affiliations:** Department of Emergency Medicine, University of Florida, 1329 SW 16th Street, P.O. Box 100186, Gainesville, FL 32610-0186, USA

## Abstract

A precipitous episode of hypotension with concomitant bradycardia is a true medical emergency especially in patients with chronic hypertension and often requires hospitalization for detailed interrogation of the underlying causes. We describe herein a case of a patient with chronic labile hypertension who presented to the ED with a sharp drop in blood pressure and heart rate which was not simply explained by an antihypertensive overdose but more so by an aggregate of the patient's multiple chronic medical conditions. This report highlights the complexities of treating simultaneous hypotension and bradycardia and the importance of discerning the underpinnings of the causes including past medical issues, patient medications, and the timeline of key events leading to the issue at hand.

## 1. Introduction

Essential hypertension is generally a medically manageable disease and regimens can often be tailor-made for each patient depending on age, gender, race and ethnicity, and other comorbid conditions. However, long standing hypertension can result in significant vascular remodeling ultimately leading to potentially life-threatening conditions such as heart attacks and strokes and end-organ damage. Among the top two leading causes of kidney transplant in the United States is long-standing and poorly or difficult to control hypertension which eventually leads to kidney failure.

In recent years, solid organ transplants have become more successful due to the improvements in surgical techniques and advancements in immunosuppressive drugs. Accordingly, the prevalence of living organ transplants is increasing even though the number of annual transplant surgeries remains constant. This selected patient population must receive careful treatment to preserve their organ transplant especially because of them many have significant comorbid condition. Immunosuppression with calcineurin inhibitors such as tacrolimus and cyclosporin are necessary to preserve renal function and prevent transplant rejection, but ironically these medications have profound nephrotoxic properties [1]. The patient discussed herein has a history of a kidney transplant and a subsequent slowly declining renal function; additionally the patient has significant hypertension which of late had been very difficult to manage.

## 2. Case Presentation

A 68-year-old man presented to the emergency department (ED) complaining of feeling lightheaded, confused, and dizzy and subsequently called for emergency medical service. En route to the ED via ambulance, the patient was noted to have a pulse in the thirties and a systolic blood pressure of 50 mmHg. Upon arrival, the patient vitals were as follows: blood pressure of 79/35, pulse of 47, oral temperature of 35.3°C, and respiratory rate of 18 with an oxygen saturation of 100 percent. His body weight was 87.3 kg, nearly 10 kg above his baseline weight. The patient was alert and oriented with a GCS of 15 but had difficulty communicating his symptoms secondary to his dizziness and feeling profoundly ill; however, he was able to clearly state that he felt he “was going to die.”

The patient's past medical history was significant for primary hypertension, type-2 diabetes mellitus, coronary artery disease, and end-stage renal disease resulting in a renal transplant five years ago. A review of the records revealed that the patient was recently hospitalized for a hypertensive emergency and pulmonary edema resultant from declining renal function. The patient's blood pressure was labile during his admission and uncontrolled during this last hospitalization. Upon discharge, the patient was told to follow up with his nephrologist to redress his hypertension.

The patient indeed followed up as instructed with his nephrologist, which was the morning of the described ED visit. At the office visit his blood pressure was noted to be 154/62. His doctor added nifedipine to his current regimen of doxazosin, hydralazine, metoprolol, and metolazone. The patient filled his new prescription and took all of the medications that afternoon except for metoprolol which he believed he was supposed to discontinue. Approximately one hour after ingesting his medications the patient became acutely ill and presented to the ED as described.

During this ED visit, in addition to the patient's abnormal vital signs and altered mental status, he was also found to be moderately hyperkalemic with potassium of 6.3 mEq/L and had an elevated BUN (40.0 mg/dL) and creatinine (2.20 mg/dL) which were both above his baseline renal function. The patient's chest X-ray was consistent with pulmonary edema and his electrocardiogram (EKG) was significant for bradycardia without hyperacute T wave or ischemic changes.

Throughout his stay in the ED, the patient received fluids, insulin and glucose, calcium gluconate, glucagon, and aerosolized albuterol in an effort to reverse his antihypertensive medications and hyperkalemia. While the patient's hyperkalemia and hypotension were corrected with treatment, he remained bradycardia while being in the ED. The patient was also warmed with warm fluids and blankets given his hypothermia. The patient was admitted for further evaluation and treatment of his fluid overload, pulmonary edema, hypotension, hyperkalemia, and bradycardia. The following diagnoses were considered: medication reaction/overdose, renal failure, endocrinopathy, and sepsis. The patient was hospitalized for almost two weeks during which he was diuresed and his blood pressure regimen was adjusted to include only doxazosin and nifedipine. Followup since discharge demonstrated the patient's hypertension to be within normal ranges.

## 3. Discussion

This case was a challenge primarily due to the patient's multiple morbidities almost all of which can contribute to the patient's presentation of hypotension or bradycardia. The challenge is identifying a plausible cause of hypotension and bradycardia. Herein, we discuss possible causes for the patient's condition and diagnosis.

### 3.1. Antihypertensive Overdose

Medication overdose was initially considered given the recent changes in the patient's regimen and moreover because the patient does not speak English which may have contributed to some confusion regarding these changes. For example, the patient noted he took all of his blood pressure medications except for Metoprolol immediately prior to his symptoms. Whereas the combination of vasodilators in his regimen can cause a sudden drop in blood pressure, the patient's bradycardia is unexplained by antihypertensive overdose since he reported he did not take metoprolol, a fact corroborated by his wife. Reflex tachycardia should follow this overdose, [1] especially since the patient's baseline systolic blood pressure is typically in 150's; thus the physiological underpinnings for bradycardia in the face of significant systemic vasodilatation are unclear. Calcium gluconate and glucagon treatment are interventions used to reverse beta blockade, [2, 3] but when these were administered his heart rate did not increase as expected, further underscoring the suggestion that beta blockade medication was not the culprit.

### 3.2. Hyperkalemia

Our patient was especially at risk for hyperkalemia given his progressively worsening renal function in the face of kidney transplant and current course of tacrolimus [4]. Hyperkalemia has been associated with first-degree AV block, atypical bundle branch blocks, and sinus arrest [5, 6] with increasing cardiac complications correlated with increasing potassium levels. We considered that hyperkalemia may have precipitated an arrhythmia thus resulting in severe bradycardia and hypotension, but the patient's EKG findings did not offer convincing evidence for this hypothesis. The typical findings of mild-to-moderate hyperkalemia (spiked T waves, widened QRS complexes, and prolonged PR interval) [6] were not present on the patient's EKG which simply showed sinus bradycardia. Additionally, it seems unlikely that the patient would have been acutely hyperkalemic compared to his chronically elevated potassium. However, these considerations do not eliminate the possibility of hyperkalemia contributing to the patient's bradycardia and hypotension. For example, Acker et al. found that less than half of patients with hyperkalemia showed typical hyperkalemia-like EKG findings [7]; this suggests lack of EKG findings alone cannot rule out hyperkalemia as a contributor of the patient's condition. Also it is noteworthy that the patient's heart rate did not increase in response to glucagon but did increase as insulin and glucose was administered. Insulin and glucose treatment is well known for shifting potassium to the intracellular compartment [8] and the fact that this treatment helped raising the patient's heart rate suggests that hyperkalemia may have played a role in slowing the patient's heart rate. Note that we did not use high dose of insulin as has been described for beta-blocker overdose.

### 3.3. Possible Hypothyroidism

As previously mentioned, the patient's temperature at the time of his ED visit was 35.3° which is considered moderately hypothermic. Moderately hypothermic patients are typically tachycardic, [9] which suggests that there was either an underlying cause behind the patient's hypothermia that lowered the patient's heart rate or that the underlying cause of this patient's presentation of hypotension, bradycardia, and hyperkalemia prevented this tachycardic response. It is usually severely bradycardia patients that present with bradycardia arrhythmias [9]. A recent report described a patient taking multiple antihypertensive drugs who presented with bradycardia, hypotension, and hypothermia, but in this case the patient did not have a change in his medications and his hypothermia was found to be secondary to myxedema coma [10]. Thyroid levels have not been obtained in the past, but they may give insight into this patient's presentation to the ED.

### 3.4. Sinus Node Dysfunction

Sinus node dysfunction should also be included in the differential for our patient: consider an elderly patient with coronary artery disease and bradycardia on the EKG. Recent research has shown that ion channels and ion currents in the sinoatrial node can undergo significant changes over time due to normal aging and that this can be exacerbated by ischemic changes brought on by coronary artery disease [11]. In our patient's case, however, there is no enough evidence to support this diagnosis since his heart rate before and after his hospitalization was well in the normal range.

Our patient was acutely ill when he presented to the ED with severe hypotension and bradycardia after a change in his hypertensive medications. We were able to resuscitate the patient by using a combination of calcium gluconate, albuterol, glucagon, and insulin with glucose. The patient had a prolonged hospitalization and upon discharge he had suffered no significant morbidity from his illness.

Ultimately, we hypothesize that our patient's condition was due to antihypertensive medications complicated by hyperkalemia. Additionally, an underlying endocrinopathy such as hypothyroidism may have to be considered to contribute to the problem.
